# A Sparse Super-Resolution Imaging Approach for Array Scanning Radar in High-Resolution Ground Mapping

**DOI:** 10.3390/s26123951

**Published:** 2026-06-22

**Authors:** Xingyu Tuo, Wen Jing, Yushi Xu, Fang Li, Bo Huang, Ge Jiang

**Affiliations:** Institute of Electronic Engineering, China Academy of Engineering Physics, Mianyang 621900, China17716847958@163.com (F.L.); vick123y@163.com (B.H.); jiangge321@163.com (G.J.)

**Keywords:** super-resolution, radar forward-looking imaging, spatial variation, sparse reconstruction

## Abstract

In airborne sensing applications, radar forward-looking imaging is a crucial technology for high-resolution ground mapping and terrain perception. Super-resolution deconvolution is key to overcoming the real-beam resolution limits of these airborne sensors. However, when utilizing phased array scanning radars for wide-swath ground mapping, the antenna pattern exhibits severe spatial variation at large scanning angles, which directly leads to model mismatch and degradation in super-resolution performance. To address this hardware-induced sensing limitation, this paper proposes a sparse super-resolution method tailored for forward-looking phased array scanning radar. Firstly, the causes of the spatial variation in antenna pattern are analyzed, and a modified antenna convolution matrix is derived to accurately model the scanning process. Secondly, the corresponding objective function is formulated under the assumption of target sparsity. Finally, an alternating direction method of multipliers (ADMM) solver based on reweighted strategy is employed to resolve the objective function. Experimental results demonstrate that the proposed method achieves approximately a 4 times increase in cross-range resolution and effectively enhances the observation capabilities within the radar forward-looking area.

## 1. Introduction

Radar forward-looking imaging serves as an indispensable sensing modality in various domains, playing a pivotal role in crucial applications such as high-resolution wide-swath ground mapping, terrain obstacle avoidance, and autonomous navigation [1,2,3]. Fundamentally, conventional monostatic synthetic aperture radar (SAR) and Doppler beam sharpening (DBS) techniques suffer from severe performance degradation in the flight direction. This limitation arises from the inherently small Doppler gradient and left-right Doppler ambiguity in the forward-looking sector, which strictly prevents high-resolution imaging along the trajectory [4,5,6,7]. To achieve forward-looking perception, existing radar imaging techniques can be broadly categorized into monopulse [8,9,10], bistatic SAR [11,12,13], and scanning radar imaging [14,15,16,17]. While monopulse radar can acquire precise angle information for an isolated target, its resolution capability sharply deteriorates in multi-target scenarios. Bistatic SAR overcomes the Doppler limitation through platform separation, yet it imposes extremely stringent requirements on time, phase, and spatial synchronization, making it cost-prohibitive for many practical deployments. Consequently, scanning radar has emerged as the most pragmatic forward-looking sensing architecture, boasting arbitrary imaging geometry and straightforward system implementation. By operating in a scanning mode, it relies on advanced signal processing to transcend the physical diffraction limits of the antenna aperture, thereby achieving forward-looking super-resolution imaging. Mathematically, the azimuth signal of a scanning radar is typically formulated as an ill-posed deconvolution problem, representing the convolution between the antenna pattern and the target scattering distribution. Driven by this formulation, various super-resolution methods have been exhaustively investigated to reconstruct targets beyond the real-beam limit [18,19]. Early attempts, such as the Lucy-Richardson algorithm [20] and improved Wiener filtering [21], provided preliminary resolution enhancements but were hindered by slow convergence or high signal-to-noise ratio (SNR) dependencies. Subsequently, regularization techniques, including truncated singular value decomposition (TSVD) [22] and Tikhonov regularization [23], were introduced to suppress noise amplification, though their resolution improvement remains bounded by the excessive smoothing of the $l_{2}$-norm penalty. Recognizing that targets of interest (e.g., specific ground structures or vehicles) typically occupy only a fraction of the illuminated scene, sparsity-driven reconstruction has become the gold standard. Researchers have successively proposed $l_{1}$-norm [24,25] and $l_{q}$-norm (q<1) [26] regularization frameworks, achieving unprecedented resolution enhancements. In addition, based on similar mathematical models, sparse methods based on spectral estimation (such as IAA [27]) have been used for radar super-resolution imaging. To further fortify robustness against noise, hybrid approaches integrating TSVD with sparse regularization have been developed, effectively mitigating noise amplification during the non-linear inversion and preserving high-resolution capabilities even under severe low-SNR conditions [28,29,30]. Recently, alongside traditional mathematical regularization schemes, data-driven frameworks such as deep learning have emerged as efficient alternatives for radar forward-looking super-resolution imaging [31,32,33]. Various neural network architectures have been investigated to learn direct mappings from low-resolution radar scans to high-resolution target distributions. Nevertheless, while these learning-based methods can effectively map standard degradation, they typically neglect the underlying physical mechanisms of real radar hardware, particularly the spatial variation in the antenna pattern.

However, a critical disconnect exists between these advanced algorithms and modern radar hardware realities. To meet the demands of high scanning rates, beam agility, and system reliability, modern airborne sensing systems have overwhelmingly transitioned from mechanical scanning to phased array architectures [34]. A fundamental physical characteristic of phased arrays is the inherent spatial variation in the antenna pattern. Specifically, the beam progressively broadens as the scanning angle steers away from the boresight. Typically, at 60° off the flight direction, the beamwidth essentially doubles [35,36].

Crucially, the aforementioned super-resolution methods predominantly operate under the ideal assumption of a shift-invariant convolution kernel, completely neglecting this spatial variation. In practical sensing applications like wide-area ground mapping, where airborne array scanning radars must continuously sweep across a vast forward-looking sector, this assumption collapses. Applying a shift-invariant algorithm to space-variant hardware data introduces severe model mismatch into the deconvolution process. This mismatch not only drastically degrades the super-resolution capability but also generates intolerable artifacts and ghost targets, severely compromising the reliability of the sensing system. To eliminate this sensing bottleneck, this paper proposes a tailored sparse super-resolution imaging method based on a reweighted strategy for forward-looking phased array scanning radar. The core contributions are as follows: Firstly, rooted in the physical principles of phased array antennas, we systematically analyze the beam broadening effect and derive a space-variant modified antenna convolution matrix to accurately model the realistic scanning process. Secondly, under the sparse regularization framework, a robust convex optimization objective function is formulated. Finally, an Alternating Direction Method of Multipliers (ADMM) solver equipped with a reweighted strategy is deployed to iteratively solve the objective function, ensuring enhanced sparsity and superior target reconstruction. The effectiveness of the proposed approach is comprehensively validated using both simulated and measured airborne radar data. The remainder of this paper is structured as follows: Section 2 derives the azimuth convolution model of the scanning radar. Section 3 details the construction of the modified antenna convolution matrix and develops the proposed reweighted sparse reconstruction methodology. Section 4 presents the simulation and experimental validation. Section 5 provides the concluding remarks.

## 2. Signal Model

**Figure 1:**
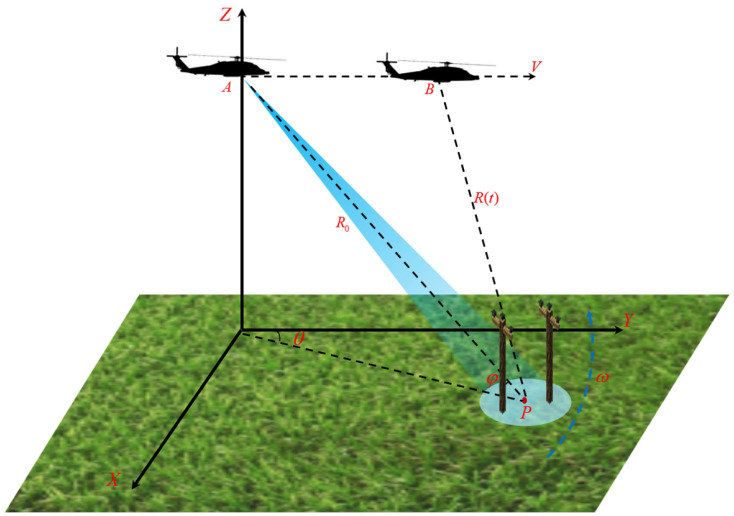
Working geometry diagram of airborne scanning radar.

It is worth noting that the imaging mode discussed in this paper is forward-looking scanning ground mapping. The airborne scanning radar transmits linear frequency modulated (LFM) pulses at a fixed frequency and achieves scan coverage over the observation area by controlling the phase. Its geometric diagram is shown in Figure 1. Assume that the initial position of the radar platform is located at point *A* and flies horizontally along the *Y* axis at a speed *V*. The equivalent scanning angular velocity of the antenna is ω. Suppose the target of interest is point target *P* in the observation area, with an initial slant range of $R_{0}$, an azimuth of θ, and an elevation angle of φ.

When the aircraft flies with interval time *t*, the radar platform arrives point *B*. The slant distance R(t) between the radar platform and the point target *P* can be expressed based on the geometric relationship as follows:

(1) $$R\left(t\right)=\sqrt{R_{0}^{2}+V^{2}t^{2}-2R_{0}Vt\cos\theta \cos\varphi }$$

For airborne forward-looking imaging, due to long initial range and fast scanning speed, Equation (1) can be approximated by Taylor’s formula as follows:

(2) $$R\left(t\right)\approx R_{0}-Vt\cos\theta \cos\varphi$$

The linear frequency modulation (LFM) signal transmitted by the scanning radar can be expressed as

(3) $$y\left(\tau \right)=\mathrm{rect}\left(\frac{\tau }{T_{p}}\right)\exp\left(j2\pi \left(f_{c}\tau +\frac{1}{2}K\tau^{2}\right)\right)$$

where τ is the radar fast time, rect(·) is the rectangular window function, $T_{p}$ is the pulse width of the transmitted signal, $f_{c}$ is the carrier frequency of the transmitted signal, and *K* is the frequency modulation ratio. The echo of point target *P* after down-conversion can be denoted as below:

(4) $$y_{p}\left(\tau ,t\right)=\sigma_{p}h\left(t\right)\;\mathrm{rect}\left(\frac{\tau -\tau_{d}}{T_{p}}\right)\exp\left(j\pi K{\left(\tau -\tau_{d}\right)}^{2}\right)\exp\left(-j2\pi f_{c}\tau_{d}\right)$$

where $\sigma_{P}$ represents the target scattering coefficient, h(t) is the antenna pattern function, $\tau_{d}=2R\left(t\right)/c$ is the echo delay, and *c* is the speed of light. Range dimension processing includes pulse compression and range motion correction, where range motion correction is performed using Equation (2) to eliminate the influence of platform movement. The echo signal after range dimension processing can be expressed as:

(5) $$y_{p}\left(\tau ,t\right)=\sigma_{p}h\left(t\right)\;\sin c\left(B\left(\tau -\frac{2R_{0}}{c}\right)\right)\exp\left(-j2\pi f_{c}\tau_{d}\right)$$

where *B* is the bandwidth of the transmitted signal, and sinc(·) is the signal envelope after pulse compression. Converting Equation (5) to the range-angle domain and considering the surface target of the entire observation scene:

(6) $$y\left(R,\theta \right)=\sigma \left(R,\theta \right)h\left(\theta -\theta_{0}\right)\;\sin c\left(\frac{2B}{c}\left(R-R_{0}\right)\right)\cdot \exp\left(-j\frac{4\pi }{\lambda }V\frac{\theta -\theta_{0}}{\omega }\right)$$

where $\exp\left(-j\frac{4\pi }{\lambda }V\frac{\theta -\theta_{0}}{w}\right)$ is the Doppler phase, which can usually be ignored on stationary or slow-moving platforms [37]. Considering the influence of additive noise, the echo signal can be modeled as the following convolution relationship:

(7) $$y\left(R,\theta \right)=\sigma \left(R,\theta \right)⊗\left\{h\left(\theta \right)\;\sin c\left(\frac{2B}{c}R\right)\right\}+n\left(R,\theta \right)$$

Equation (7) illustrates that the echo signal of the scanning radar can be modeled as a two-dimensional separable convolution process. For the range dimension, it represents the convolution of the target scattering distribution with the pulse compression response function. Thus, range resolution can be enhanced by transmitting a large-bandwidth signal. For the azimuth dimension, the signal is modeled as the convolution of the target scattering distribution with the antenna pattern. Azimuth resolution can be improved using deconvolution methods.

Extracting azimuth dimension signal, Equation (7) can be further simplified as

(8) y(θ)=σ(θ)⊗h(θ)+n(θ)

## 3. Array Scanning Radar Sparse Super-Resolution Method

### 3.1. Analysis of Spatial Variability of Array Antenna Patterns

Firstly, the spatial variation in antenna pattern for phased array scanning radar is analysed. Considering a one-dimensional uniform linear array with *M* array elements, according to the array pattern principle, the 3db width of the main lobe of the array pattern (named as $\theta_{B}$) can be expressed as

(9) $$\theta_{B}\approx \frac{0.886\lambda }{Md}\frac{1}{\cos\theta }$$

where λ represents the wavelength, *M* is the number of array elements, *d* is the array element spacing, and θ is the beam scanning angle. From this formula, it can be seen that the beam width $\theta_{B}$ varies with the scanning angle θ. Typically, when the scanning angle θ=60°, the beam width $\theta_{B}$ doubles.

### 3.2. Correcting the Antenna Convolution Matrix

From Equation (8), we see that for the azimuth echo, it can be expressed as the convolution of the target’s azimuth scattering distribution and the antenna pattern when the Doppler frequency shift is ignored. The azimuth echo in Equation (8) can be rewritten in matrix form as:

(10) $$y_{N\times 1}=H_{N\times M}\sigma_{M\times 1}+n_{N\times 1}$$

where $y_{N\times 1}$ represents the azimuth echo signal vector received by the scanning radar, $H_{N\times M}$ represents the antenna convolution matrix, $\sigma_{M\times 1}$ represents the target scattering distribution vector in the same range cell, and $n_{N\times 1}$ represents the noise vector. Among them, *N* is the number of sampling points of the azimuth echo signal, and *M* is the number of sampling points of the scanning area. The antenna convolution matrix is constructed by cyclically shifting the antenna pattern sampling vector. Ignoring the spatial variation in antenna pattern, the ideal antenna convolution matrix can be expressed as

(11) $$H_{N\times M}=\left[\begin{array}{ccccccc} h_{1} & h_{2} & \cdots & h_{L} & & & \\ & h_{1} & h_{2} & \cdots & h_{L} & & \\ & \; & \ddots & \ddots & \ddots & \ddots & \\ & \; & & h_{1} & h_{2} & \cdots & h_{L} \end{array}\right]$$

where $\left[\begin{array}{cccc} h_{1} & h_{2} & \cdots & h_{L} \end{array}\right]$ is the sampling vector of the antenna pattern, *L* is the number of sampling points of the antenna pattern, and its value is determined by the beam width $\theta_{B}$ and beam position step size. According to the formula, $L=\theta_{B}\cdot PRF/\omega$, ω is the equivalent scanning speed, and PRF represents the pulse repetition frequency. $H_{N\times M}$ has *N* rows, where the *i*-th row represents the sampled antenna pattern during the *i*-th scanning angle. Since the ideal antenna convolution matrix ignores the spatial variation in the pattern, the number of effective sampling points *L* remains constant across the entire scanning field. From the previous analysis, it can be concluded that the beam width $\theta_{B}$ of the phased array radar varies with the scan angle θ. This variation in beam width $\theta_{B}$ directly affects the number of sampling points of the antenna pattern Lfx, consequently influencing the construction of the antenna convolution matrix. Specifically, the phased array antenna exhibits distinct beam broadening characteristics at different scan angles, resulting in variable numbers of antenna pattern sampling points. This relationship can be mathematically expressed as

(12) $$L\left(\theta_{i}\right)=\frac{0.886\lambda }{Md}\frac{PRF}{\cos\theta_{i}\cdot \omega }$$

where $\theta_{i}=\left[\theta_{1},\theta_{2},\cdots \theta_{N}\right],\theta_{1},\theta_{2},\cdots \theta_{N}$ represents the discrete sampling of the scanning angle. Based on the construction principle of the antenna convolution matrix, the ideal antenna convolution matrix can be corrected to the following expression:

(13) $${\hat{H}}_{N\times M}=\left[\begin{array}{ccccccc} h_{1,1} & h_{1,2} & \cdots & h_{1,L(\theta_{1})} & & & \\ & h_{2,1} & h_{2,2} & \cdots & h_{2,L(\theta_{1})} & & \\ & \; & \ddots & \ddots & \ddots & \ddots & \\ & \; & & h_{N,1} & h_{N,2} & \cdots & h_{N,L(\theta_{N})} \end{array}\right]$$

where $h_{i,j}$ represents the *j*-th element value of the antenna pattern sampling vector at the *i*-th scan. Specifically, $\left[\begin{array}{cccc} h_{1,1} & h_{1,2} & \cdots & h_{1,L(\theta_{1})} \end{array}\right]$ is the sampling vector of the antenna pattern during the first scan angle $\theta_{1}$, and $L(\theta_{1})$ is the corresponding number of antenna pattern sampling points. Similarly, $\left[\begin{array}{cccc} h_{2,1} & h_{2,2} & \cdots & h_{2,L(\theta_{2})} \end{array}\right]$ and $\left[\begin{array}{cccc} h_{N,1} & h_{N,2} & \cdots & h_{N,L(\theta_{N})} \end{array}\right]$ are shown in the same way. Compared with the ideal antenna convolution matrix, the modified version in Equation (13) incorporates angle-dependent beamwidth $\theta_{B}/\cos\theta_{i}$ at each scan angle. Consequently, the number of sampling points per row is no longer a constant value, but instead varies with the scanning angle $\theta_{i}$. This formulation accounts for the spatial variation in antenna pattern, providing more accurate characterization of the convolution process over scanning regions. Specifically, Equation (13) better reflects the physical reality of phased array antennas during large-range scanning operations.

### 3.3. Sparse Method Based on Reweighted ADMM

Using the above modified antenna convolution matrix, the azimuth echo model in Equation (10) can be transformed into:

(14) $$y_{N\times 1}={\hat{H}}_{N\times M}\sigma_{M\times 1}+n_{N\times 1}$$

In radar imaging applications, it is commonly assumed that the targets of interest exhibit sparse distributions. Specifically, the number of target sampling points is significantly smaller than the total sampling points of the scanning scene, and the target scattering intensity substantially exceeds the average scattering coefficient of other regions in the scanning scene. Typical examples include ship targets on sea surfaces and military vehicle targets on ground terrains. By incorporating this sparse prior of targets, the following objective function can be derived under the regularization framework:

(15) $$\sigma =\arg\min_{\sigma }{∥y-\hat{H}\sigma ∥}_{2}^{2}+\lambda {∥\sigma ∥}_{1}$$

The $l_{1}$ norm is non-differentiable at the origin, necessitating specialized optimization techniques to solve the problem in Equation (15). Common approaches include: the ADMM, the Fast Iterative Shrinkage-Thresholding Algorithm (FISTA), and the Iterative Reweighted Norm (IRN) method. Among these, the ADMM algorithm demonstrates superior computational efficiency, reliable convergence properties, and excellent scalability for large-scale parallel computing [38], making it our preferred solver. To enhance target sparsity, we propose a reweighted-ADMM approach that substitutes the standard $l_{1}$ norm with a weighted $l_{1}$ norm. The method iteratively updates the weight matrix after solving each weighted $l_{1}$ minimization subproblem, thereby achieving sparser solutions. The specific solution steps are summarized as follows:

Initialization:

(16) $$\sigma^{\left(0\right)}={\left({\hat{H}}^{T}\hat{H}+\lambda I\right)}^{-1}{\hat{H}}^{T}y$$

Among them, λ represents the regularization parameter, which is used to balance the strength of the regularization constraint term and the noise term, and can be selected by the L-curve method [39]. This method identifies the corner point of the parametric curve of the log-residual norm versus the log-solution norm to yield a robust trade-off.

Iteration:

a. Calculate the weight matrix:

(17) $$W^{\left(k\right)}=diag\left(\frac{1}{\sigma^{\left(k-1\right)}+\delta }\right)$$

Among them, δ is a small positive number added to avoid zero values, usually $\delta =1\times 10^{-6}$.

b. Solve the weighted $l_{1}$ norm minimization problem:

(18) $$\sigma^{\left(k\right)}=\arg\min_{\sigma^{\left(k\right)}}{∥y-\hat{H}\sigma^{\left(k\right)}∥}_{2}^{2}+\lambda {∥W^{\left(k\right)}\sigma^{\left(k\right)}∥}_{1}$$

Equation (18) formulates the weighted $l_{1}$ norm minimization problem. In this step, the alternating direction multiplier method (ADMM) is used to resolve it. Let the inner-loop iteration sequence be denoted as *j*, the ADMM implementation for weighted $l_{1}$ norm minimization consists of the following steps:

(19) $$\begin{array}{cc} & \sigma^{\left(k\right)}={\left[{\hat{H}}^{T}\hat{H}+p{\left(W^{\left(k\right)}\right)}^{T}\left(W^{\left(k\right)}\right)\right]}^{-1}\left[{\hat{H}}^{T}y+p{\left(W^{\left(k\right)}\right)}^{T}\left(z-u\right)\right]\\ & z=S_{\frac{\lambda }{p}}\left(u+W^{\left(k\right)}\sigma^{\left(k\right)}\right)\\ & u=u+p(W^{\left(k\right)}\sigma^{\left(k\right)}-z) \end{array}$$

where *p* denotes the Lagrangian penalty parameter, typically selected within the range 1∼10. The inner loop terminates when the error between consecutive iterations satisfies the predefined threshold condition η. The specific convergence criterion is mathematically expressed as

(20) $$\frac{{∥\sigma_{\;j+1}^{\left(k\right)}-\sigma_{\;j}^{\left(k\right)}∥}_{2}}{{∥\sigma_{\;j+1}^{\left(k\right)}∥}_{2}}\leq \eta =0.001$$

c. Update outer loop:

(21) k=k+1

Through iterative computation of Equations (17) and (19), the proposed method progressively approximates the true values of target scattering coefficients, ultimately obtaining a sparse reconstruction. The incorporation of the reweighted strategy ensures rapid convergence in the outer loop, such that global convergence is guaranteed when the outer loop iteration count satisfies k=15 [40].

## 4. Simulation Verification

To validate the performance of the proposed method, we conduct comprehensive simulations focusing on both point targets and experimental data. The evaluation compares our approach with conventional super-resolution techniques, including the Tikhonov super-resolution method [23], the sparse super-resolution method [17], and the Iterative Adaptive Approach (IAA) super-resolution method [27].

### 4.1. Point Target Processing Results

This subsection evaluates the imaging performance of the proposed method using one-dimensional point targets. The simulation parameters and environment are detailed in Table 1 and Table 2, respectively. The signal-to-noise ratio (SNR) is set to 20 dB and the regularization parameter λ is set to 0.01 based on the L-curve method. Six point targets are set, all with an equivalent target scattering coefficient (RCS) of 1, and positioned at ±2.5°, ±25.5°, and ±30.5°.

**Figure 2:**
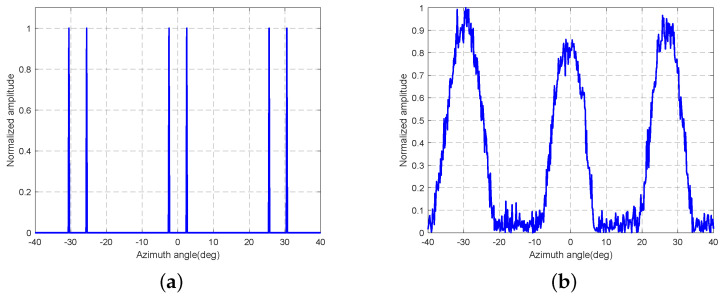

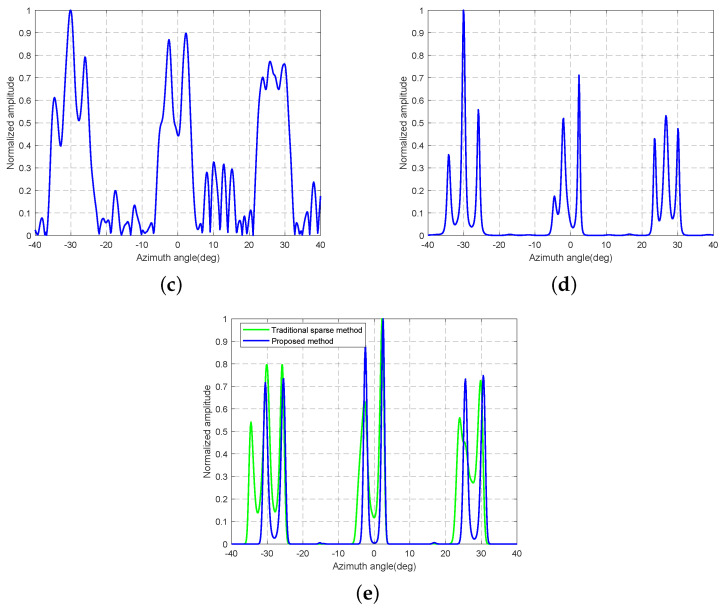
Point targets processing results: (**a**) Target distribution; (**b**) Real beam echo; (**c**) Tikhonov super-resolution method; (**d**) IAA super-resolution method; (**e**) The proposed method.

Figure 2 presents the point target processing results. Figure 2a shows the original target scene with six sparsely distributed point targets. Figure 2b is the real beam echo after range dimension processing, where target echoes are aliased due to the target spacing being less than the antenna beam width. Figure 2c shows the result of the traditional Tikhonov super-resolution method, which achieves limited resolution improvement. Figure 2d shows the result of the IAA super-resolution method. Although the resolution improvement is significant, the lack of consideration for spatial variation in antenna pattern produces false peaks. Figure 2e shows the comparison results between the traditional sparse method and the proposed method. Since the variation in antenna patterns is ignored, the traditional sparse method shows high resolution but has obvious false peaks on the left. The proposed method effectively reconstructs the six sparse point targets, taking into account both antenna pattern variation and target sparsity, thereby avoiding false peaks.

In summary, compared to real beam echo, our proposed method effectively improves azimuth resolution and resolves adjacent aliased targets. Compared to traditional sparse super-resolution methods, our proposed method avoids false targets and achieves effective reconstruction of sparse targets by accounting for antenna pattern variation.

### 4.2. Comparison of Super-Resolution Performance at Different Signal-to-Noise Ratios

This subsection further assesses the effectiveness of the proposed method by setting different signal-to-noise ratios. For simplicity, only the traditional sparse method and the proposed sparse method are compared, with simulation parameters and scenarios consistent with those used in Section 4.1. To quantitatively evaluate the processing results, two metrics are introduced: relative error (Re Err) and structural similarity index (SSIM) [41]. The relative error is defined as:

(22) $$\mathrm{ReErr}=\frac{{∥\hat{\sigma }-\sigma ∥}_{2}}{{∥\sigma ∥}_{2}}$$

Among them, σ and $\hat{\sigma }$ represent the true target scattering coefficient and the solved target scattering coefficient respectively. A smaller relative error indicates that the solved target scattering coefficient is closer to the true value, thereby demonstrating better super-resolution performance. Structural similarity is defined as:

(23) $$\mathrm{SSIM}=\frac{2\rho \left(\hat{\sigma },\sigma \right)\cdot \left(2u_{\hat{\sigma }}\cdot u_{\sigma }\right)}{\left(u_{\hat{\sigma }}^{2}+u_{\sigma }^{2}\right)\left(v_{\hat{\sigma }}^{2}+v_{\sigma }^{2}\right)}$$

Among them, *u* and *v* are the mean and standard deviation of the vectors σ and $\hat{\sigma }$ respectively. $\rho (\hat{\sigma },\sigma )$ is the correlation coefficient of the vectors σ and $\hat{\sigma }$. The structural similarity reflects the similarity between the solved target scattering coefficient and the true target scattering coefficient. The larger its value, the better the super-resolution performance.

**Figure 3:**
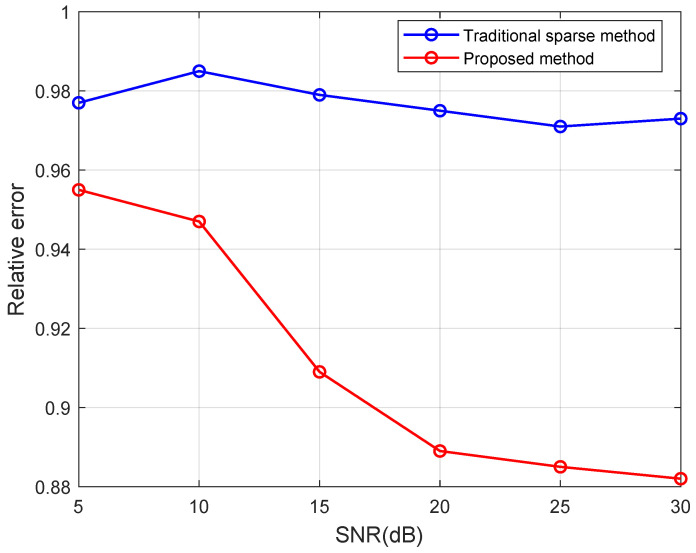
Relative error curve under different signal-to-noise ratios.

**Figure 4:**
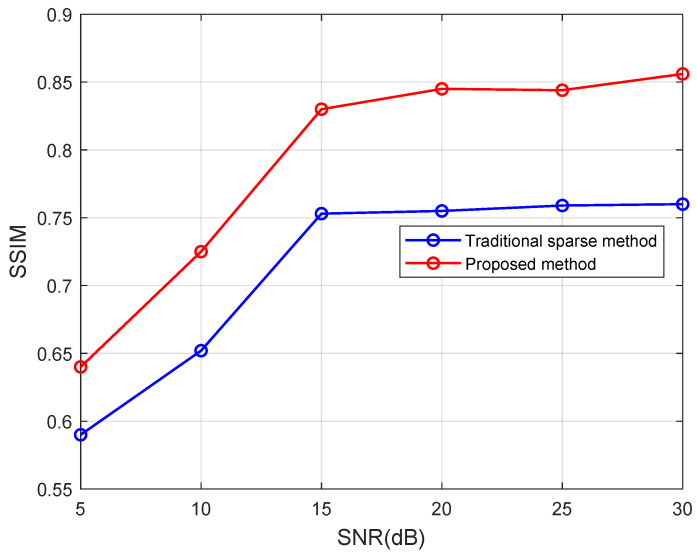
Structure similarity curve under different signal-to-noise ratios.

From the relative error curves shown in Figure 3, it can be observed that the relative errors of the two methods generally show a decreasing trend as the SNR increases. The relative error of the traditional sparse method is always significantly greater than that of the proposed method. This is because the proposed method takes into account the variation in the antenna pattern and avoids the introduction of errors in the deconvolution procedure. Figure 4 shows that the SSIM values of the two methods gradually rises as the SNR increases. The structural similarity of the proposed method is consistently better than that of the traditional sparse method, further demonstrating the superiority of the proposed method.

### 4.3. Experimental Processing Results

The effectiveness of the proposed method has been demonstrated through point target simulation. Now, we will further verify the effectiveness of the proposed method using experimental data. This experimental data consists of two groups: the first group is stationary platform experimental data and the second group is airborne platform experimental data. Crucially, while full-reference metrics such as ReErr and SSIM index are well-suited for simulations where absolute true values are available, they are not applicable to measured data due to the lack of real target scattering coefficients. Therefore, this paper introduces no-reference image quality assessment criteria, specifically image contrast (IC) and image entropy (IE) [42], to objectively evaluate focus quality and artifact suppression capabilities on measured data.

IC reflects the clarity of the target against the background clutter, which is mathematically defined as the ratio of the standard deviation of the image amplitude to its mean value:

(24) $$C=\frac{\sqrt{\frac{1}{MN}\sum_{m=1}^{M}\sum_{n=1}^{N}{\left(|X(m,n)|-\mu \right)}^{2}}}{\mu }$$

where X(m,n) represents the scattering coefficient at the spatial coordinate (m,n), *M* and *N* represent the total number of range and azimuth cells in the radar image, respectively, and μ denotes the mean amplitude of the entire image. Crucially, to avoid the non-linear distortion effects induced by logarithmic compression, both the IC and IE in this paper are calculated directly based on the linear amplitude values of the reconstructed radar image matrix X(m,n), rather than on its decibel (dB) representation. A higher contrast value indicates that the target features are more distinctly separated from the background, reflecting superior super-resolution and clutter suppression performance.

IE evaluates the statistical order and focusing quality of the reconstructed radar image. Its calculation formula is defined as:

(25) $$E=-\sum_{m=1}^{M}\sum_{n=1}^{N}p\left[|X\left(m,n\right)|\right]\ln p\left[|X\left(m,n\right)|\right]$$

where p[|X(m,n)|] represents the distribution probability of the target’s amplitude at (m,n), reflecting the statistical distribution of the scattering coefficient across the entire image. Fundamentally, the essence of super-resolution imaging is to eliminate the azimuth “blur” induced by the antenna pattern. From an information-theoretic perspective, this reconstruction is a typical entropy reduction process. Therefore, superior super-resolution performance intrinsically yields a smaller entropy in the reconstructed image.

- **Stationary platform experiment**

**Figure 5:**
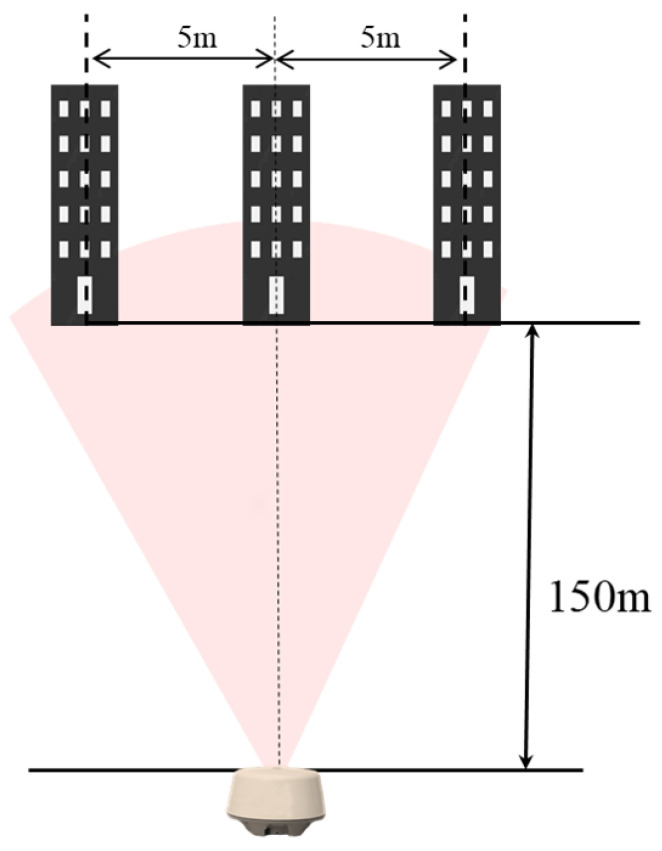
Experimental scenario diagram.

The schematic diagram of the experimental scenario of the first group is shown in Figure 5. There are three buildings in the observation area in front of the radar. The three buildings are located in the same range cell. The radar covers the observation area through an array scanning beam. The radar system parameters for the stationary platform experiment are as follows, see Table 3. Based on the L-curve selection under close-range conditions, the regularization parameter λ is set to 0.12 for this stationary scenario.

The distance between buildings is about 5 m, the distance between the radar and the buildings is about 150 m, the beam width of the radar is 8 degrees, and the real beam resolution of the radar is calculated to be:

(26) $$\Delta \rho =R\theta =150\times \frac{8\pi }{180}\approx 20.94$$

**Figure 6:**
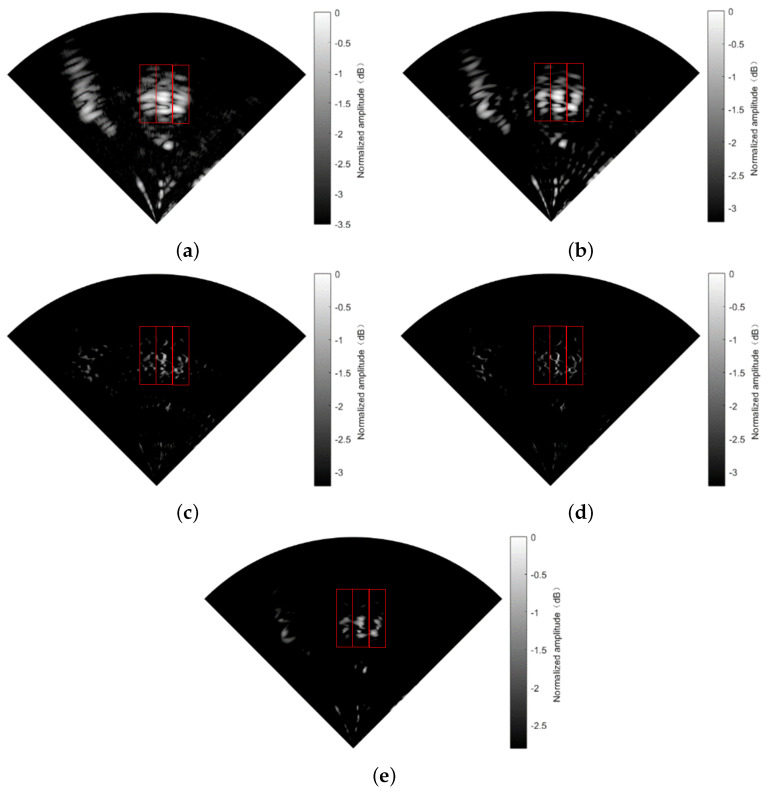
The first group of experimental data processing results (where the red bounding boxes across all subfigures outline the ground-truth physical boundary zones corresponding to the three target buildings for comparison): (**a**) Real beam echo; (**b**) Tikhonov super-resolution method; (**c**) Sparse super-resolution method; (**d**) IAA super-resolution method; (**e**) The proposed method.

As can be seen from the real beam resolution, without super-resolution technology, the radar can only resolve targets with an cross-range spacing of approximately 20 m. The three buildings will overlap, so super-resolution technology is needed to improve its cross-range resolution. Figure 6 presents the processing results of the experimental data. Figure 6a shows the real beam echo result. Due to the limitation of the antenna aperture, the real beam resolution is poor, and the three houses cannot be distinguished. Figure 6b displays the result of the traditional Tikhonov super-resolution method. The resolution improvement is insufficient, resulting in the three houses not being completely separated. Figure 6c,d present the results of the traditional sparse method and the IAA method, respectively. Although the resolution is high, the results contain many false strong points due to the neglect of antenna pattern variation, making it impossible to determine the state of the forward-looking area. Figure 6e shows the result of the proposed method, it is clearly visible that there are three targets ahead.

From the processing results of the first group of experimental data, we can see that the proposed method can distinguish three adjacent building targets, achieving a resolution improvement of about 20.94/5=4.188 times.

- **Airborne platform experiment**

**Figure 7:**
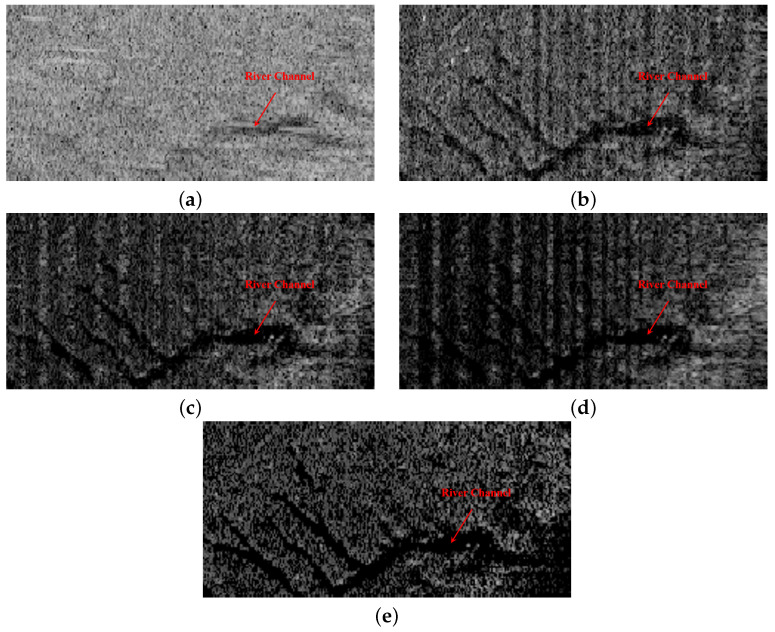
The second group of experimental data processing results: (**a**) Real beam echo; (**b**) Tikhonov super-resolution method; (**c**) Sparse super-resolution method; (**d**) IAA super-resolution method; (**e**) The proposed method.

The second group of experimental data comes from the airborne platform. The airborne radar collects echo data of the observation area through array beam scanning. The carrier frequency of the airborne radar is X-band, the scanning speed is 5°/s, the pulse repetition interval is 512 us, and the beam width is 2.2°. The experimental data processing results are shown in Figure 7. To fortify noise suppression, the regularization parameter λ is set to 0.58 for the river channel terrain reconstruction. In Figure 7, the prominent river channel features are explicitly marked with callout arrows to guide the reader. Structurally, although a natural river terrain represents a continuously distributed scene rather than isolated points, it inherently satisfies the sparse sensing representation in high-resolution radar mapping. Specifically, the smooth water surface acts as a specular reflector yielding minimal backscattering (forming the dark low-energy zones), whereas the complex land-water boundaries and riverbanks produce strong, distinct localized scattering centers. This pronounced “dark background vs. bright boundaries” contrast endows the terrain profile with strong spatial sparsity.

Figure 7a displays the real beam result after pulse compression and range motion correction. Due to the poor resolution of the real beam, it is difficult to obtain effective information about the observation area. Figure 7b presents the result of the traditional Tikhonov super-resolution method. Due to the limited resolution improvement, only rough river channel information can be observed, but details are blurred. Figure 7c,d show the results of the traditional sparse method and the IAA method, respectively. Although the resolution is high, the neglect of antenna pattern variation results in noticeable “vertical stripes,” which degrades imaging quality. Figure 7e illustrates the processing result of the proposed method. The details of the river channel are clearly observed, and the generation of “vertical stripes” is avoided, demonstrating a significant improvement in imaging quality compared to traditional methods. The processing results of the second group of experimental data confirm the effectiveness of the proposed method in practical applications. It significantly enhances the quality of super-resolution imaging and improves the radar’s perception capabilities for targets in the forward-looking area.

To further validate the visual improvements observed in Figure 6 and Figure 7, Table 4 provides a comprehensive quantitative performance comparison of the experimental ground mapping data.

As quantitatively demonstrated in Table 4, the proposed method achieves the highest image contrast in the stationary platform scenario. This numerical advantage perfectly aligns with the visual result that our method can distinctly resolve the three adjacent building targets from background clutter. Furthermore, in the complex airborne river channel mapping scenario, the proposed method yields the lowest image entropy. This strictly confirms that by compensating for the spatial variation in the antenna pattern, the proposed method significantly suppresses false peaks and “vertical stripe” artifacts, thereby concentrating the target energy much more effectively than the Tikhonov and traditional sparse methods. Ultimately, both metrics corroborate the proposed method’s superiority in high-resolution terrain perception.

## 5. Conclusions

In this paper, a tailored sparse super-resolution imaging approach is proposed to overcome the inherent hardware limitations of phased array scanning radars in high-resolution ground mapping. Recognizing that severe beam broadening at large scanning angles causes inevitable model mismatch in traditional shift-invariant algorithms, we formulated a space-variant modified antenna convolution matrix to accurately characterise the physical scanning process. By integrating this precise sensing model with a reweighted Alternating Direction Method of Multipliers (ADMM) framework, the proposed method effectively reconstructs sparse targets while rigorously suppressing noise amplification. Comprehensive evaluations using both simulated and measured airborne radar data validate the superiority of the proposed approach. Visual and quantitative results demonstrate that our method achieves an approximately four-fold increase in cross-range resolution. More importantly, by yielding the lowest image entropy and the highest image contrast compared to conventional Tikhonov and unweighted sparse methods, our research enhances the forward-looking perception capabilities of modern airborne radar systems in complex terrain mapping scenarios.

## Figures and Tables

**Table 1:** Simulation parameters.

Parameter	Parameter Value
Carrier frequency	10.75 GHz
Signal bandwidth	80 MHz
Signal pulse width	$2\times 10^{-6}$ s
Pulse repetition frequency	500 Hz
Antenna scanning speed	80°/s
Beam width	5°
Scanning range	−40°∼+40°

**Table 2:** Simulation conditions.

Parameter	Parameter Value
CPU	Intel(R) Core(TM) i7-9700K
RAM	16 GB
Simulation Software	Matlab 2022a

**Table 3:** Radar system parameters.

Parameter	Parameter Value
Carrier frequency	10 GHz
Signal bandwidth	75 MHz
Signal pulse width	$2\times 10^{-6}$ s
Pulse repetition frequency	200 Hz
Antenna scanning speed	75°/s
Beam width	8°
Scanning range	−45°∼+45°

**Table 4:** Quantitative performance comparison of experimental ground mapping data.

Scenario	Metric	Tikhonov	Traditional Sparse	Proposed Method
Stationary Platform	Image Contrast	12.34	17.56	**25.42**
Airborne Platform	Image Entropy	5.12	4.88	**2.95**

The bold values in the table highlight the optimal quantitative metric in each scenario (highest contrast for stationary, lowest entropy for airborne platform).

## Data Availability

The data supporting the reported results are available from the corresponding author upon reasonable academic request.
